# Ensemble Learning Improves the Efficiency of Microseismic Signal Classification in Landslide Seismic Monitoring

**DOI:** 10.3390/s24154892

**Published:** 2024-07-28

**Authors:** Bingyu Xin, Zhiyong Huang, Shijie Huang, Liang Feng

**Affiliations:** 1 Faculty of Resource and Environmental Engineering, Jiangxi University of Science and Technology, Ganzhou 341000, China; xinby07@163.com; 2 Institute of Mountain Hazards and Environment, Chinese Academy of Sciences, Chengdu 610041, China; zhiyongh041@gmail.com; 3 University of Chinese Academy of Sciences, Beijing 100049, China; 4 School of Computer Science and Technology, Xinjiang University, Urumqi 830017, China; 107552101298@stu.xju.edu.cn; 5 Jiangxi Provincial Key Laboratory of Environmental Pollution Prevention and Control in Mining and Metallurgy, Ganzhou 341000, China

**Keywords:** microseismic monitoring, machine learning, ensemble learning, signal identification

## Abstract

A deep-seated landslide could release numerous microseismic signals from creep-slip movement, which includes a rock-soil slip from the slope surface and a rock-soil shear rupture in the subsurface. Machine learning can effectively enhance the classification of microseismic signals in landslide seismic monitoring and interpret the mechanical processes of landslide motion. In this paper, eight sets of triaxial seismic sensors were deployed inside the deep-seated landslide, Jiuxianping, China, and a large number of microseismic signals related to the slope movement were obtained through 1-year-long continuous monitoring. All the data were passed through the seismic event identification mode, the ratio of the long-time average and short-time average. We selected 11 days of data, manually classified 4131 data into eight categories, and created a microseismic event database. Classical machine learning algorithms and ensemble learning algorithms were tested in this paper. In order to evaluate the seismic event classification performance of each algorithmic model, we evaluated the proposed algorithms through the dimensions of the accuracy, precision, and recall of each model. The validation results demonstrated that the best performing decision tree algorithm among the classical machine learning algorithms had an accuracy of 88.75%, while the ensemble algorithms, including random forest, Gradient Boosting Trees, Extreme Gradient Boosting, and Light Gradient Boosting Machine, had an accuracy range from 93.5% to 94.2% and also achieved better results in the combined evaluation of the precision, recall, and F1 score. The specific classification tests for each microseismic event category showed the same results. The results suggested that the ensemble learning algorithms show better results compared to the classical machine learning algorithms.

## 1. Introduction

A landslide is a kind of geologic hazard that arises when the stability of a slope is compromised, primarily in a mountainous area. It poses a significant peril to both the natural environment and human life [1]. According to the National Bureau of Statistics of China, an annual average of 8961 geological disasters, 6148 landslides, and 1807 avalanches occurred from 2012 to 2021 in China. During that decade, there were 4550 fatalities and direct economic losses of 4,446,191,000 CNY caused by geological disasters. Landslides predominantly occur in mountainous terrains, inclines within hilly regions, riverbanks, artificial embankments, and excavations, among other vulnerable areas [2]. They pose a threat to engineering and construction projects. In the case of minor disasters, they can impact building construction; however, in more severe cases, they have the potential to cause significant damage to buildings, disrupt traffic flow, and hinder normal road transport. Moreover, large-scale landslides can lead to river blockages, destruction of roads and infrastructure facilities such as factories and mines, as well as the burial of villages. The economic repercussions resulting from these events are challenging to quantify accurately. To enhance the safety of individuals residing near disaster-prone sites, further measures such as monitoring systems and early warning mechanisms for hazardous slopes have been proposed with increasingly stringent requirements.

Landslides and rock slides exhibit brittle, destructive behavior, releasing energy during their deformation and destruction processes. This phenomenon generates elastic waves that propagate through solid media, with seismometers capable of capturing the location of their generation and the magnitude of the released energy [3]. Microseismic monitoring is a technique specifically designed to capture micro rupture signals [4]. In recent years, microseismic monitoring technology has played an increasingly significant role in the development of landslide monitoring. In the early warning detection of geologic hazards in the slope environment, a variety of equipment such as geo-radar, inclinometers, seismometers, etc., can be utilized for the real-time monitoring of landslides and collapses. Variations in microseismic signals are analyzed to reflect geohazard phenomena [5]. Signal monitoring is typically accomplished using instruments that ensure uninterrupted 24 h surveillance, thereby providing ample data for subsequent processing. The available microseismic instruments not only capture heterogeneous signals generated naturally but also record signals originating from anthropogenic activities. Given that man-made sources such as human activities can introduce interference when analyzing source activity, it becomes imperative to classify the sources of microseismic signals as an initial step in signal processing analysis.

The waveform recognition and automatic processing can be roughly divided into two main steps: (1) microseismic event arrival-time detection and (2) event classification. There are numerous research methods both domestically and internationally for arrival-time detection: (1) The short-term average (STA)/long-term average (LTA) method [6] is used to reflect the variations in the signal amplitude and frequency. When an abnormal event occurs, the value of the STA/LTA will be changed abruptly. By observing their ratios, it is possible to determine when microseismic events occur and to identify them from background noise. However, this method heavily relies on selecting an appropriate threshold value, making it crucial to carefully choose the suitable threshold value during practical applications. (2) Intercorrelation [7] is when a signal is convolved with another signal, incorporating a specific time delay, resulting in the generation of an intercorrelation function. The magnitude of the mutual correlation function directly corresponds to the level of similarity between the two signals. This technique is employed for comparing consecutive signal sequences to identify anomalous variations, specifically pinpointing the temporal occurrence of microseismic events. Other commonly utilized methodologies include template matching, wavelet transform, and the Akaike information criterion (AIC) [8], among others.

Feng et al. [1] conducted a comparative analysis of the STA/LTA method and intercorrelation method for detecting microseismic event arrivals. They applied these two methods to analyze a short signal trajectory consisting of two strong events and three weak events while also discussing the appropriate detection thresholds. The results of their experiment revealed that the intercorrelation method is susceptible to environmental noise. Therefore, it is more suitable for stable monitoring environments or when dealing with filtered signals and high signal-to-noise data, as well as for single-event detection purposes. The template matching method [9] focuses on collecting enough templates for microseismic events, and the accuracy of the recognition depends on the number of templates used in the training process. Because templates are susceptible to signal sources, different environments may have different effects on the trained model, thus affecting the robustness and portability of the model. The microseismic signals discussed in this paper are derived from the natural state in the field, where human activities and meteorological and geological environments are subject to a certain degree of variability, which puts higher requirements on the detection methods. Therefore, this paper adopts the model based on the STA/LTA method for detection.

The use of machine learning algorithms in geoscience data processing has increased with the development of artificial intelligence technology [10,11,12,13]. Their powerful generalization ability has been demonstrated in several practical applications. Currently, experts and scholars worldwide have conducted extensive research on landslide interpretation, landslide susceptibility evaluation, and other fields using machine learning. Integrating remote sensing technology, the big data cloud platform, and engineering application processing technology enables the implementation of a machine learning algorithm to identify potential landslide hazards. This approach establishes a high-precision intelligent identification model, which is of great practical significance for disaster prevention and mitigation.

Meanwhile, scholars have conducted research on classifying microseismic events using machine learning. Long et al. [14] analyzed the differences in waveform parameter characteristics of typical signals acquired by the microseismic system from the Asher copper mine. They proposed a method of identifying rock rupture signals based on the decision tree (DT) classification algorithm and carried out a comparative analysis of its identification accuracy. The research results reveal that the distribution ranges of parameters overlap to differing extents. Therefore, it is not possible to effectively identify rock rupture signals using a single parameter. To eliminate the influence of noise signals, a DT classification model was used to construct a rock body rupture signal recognition model. This model effectively eliminates the influence of noise signals, resulting in a recognition accuracy rate of 97.8%. This rate is significantly higher than that of the Support Vector Machine model, which is 73.9%. Provost et al. [15] used a random forest (RF) classifier based on seismic attributes. The proposal includes 71 effective seismic attributes that can represent microseismic events. Models were developed to detect four types of seismic sources on a seismic network of eight sensors located on the Super-Sauze clay-rich landslide in the Southern Alps of France. The model achieved a sensitivity of up to 93% when compared to the manually interpreted catalog used as a reference. Based on this, Langet [16] utilized a convolutional neural network (CNN) to automatically classify 15 years of seismic signals recorded by a network of eight geophones installed around the steep slopes behind the rocky slopes of Aknes, Norway. The classifier’s performance was estimated to be close to 80%. Malfante et al. [17] investigated the Ubinaus volcano acquisition of 109,434 volcanic seismic events, constructed a new model based on Support Vector Machine (SVM), and achieved a correct classification rate of 92. Maggi et al. [18] distinguished eight categories of signals based on microseismic data acquired by the Piton de la Fournaise Volcanic Observatory: summit and deep volcanic tectonic events, local, regional, teleseismic, T-phase, rockfall, and sonic. They constructed a classifier using an RF classifier and achieved a classification accuracy of 96%. The study did not include subjective evaluations. Peng et al. [19] used ten machine learning algorithms to establish the discrimination of microseismic events and blasts, including DT, RF, logistic regression (LR), SVM, K-nearest neighbor (KNN), Gradient Boosting Trees (GB), Naive Bayes (NB), Bagging, AdaBoost, and Multi-layer Perceptron classification (MLP). The results showed that LR had the best performance in parameter identification, and the accuracy of cross-validation can reach more than 0.95.

This paper compared different machine learning models on the microseismic event dataset created from the Jiuxianping landslide in Yunyang, Chongqing. The aim was to identify the models with the highest classification accuracy and efficiency. Firstly, a category dataset was established to evaluate the strengths and weaknesses of the algorithms. The dataset contained feature parameters, such as the maximum amplitude, maximum frequency, mean-to-peak ratio, duration, and center frequency. At the same time, the microseismic events were classified into categories according to specific rules. Secondly, machine learning models were built to classify the microseismic events. Finally, the performance of each model was analyzed based on the experimental results, and their advantages and disadvantages were summarized.

## 2. Dataset

**Figure 1:**
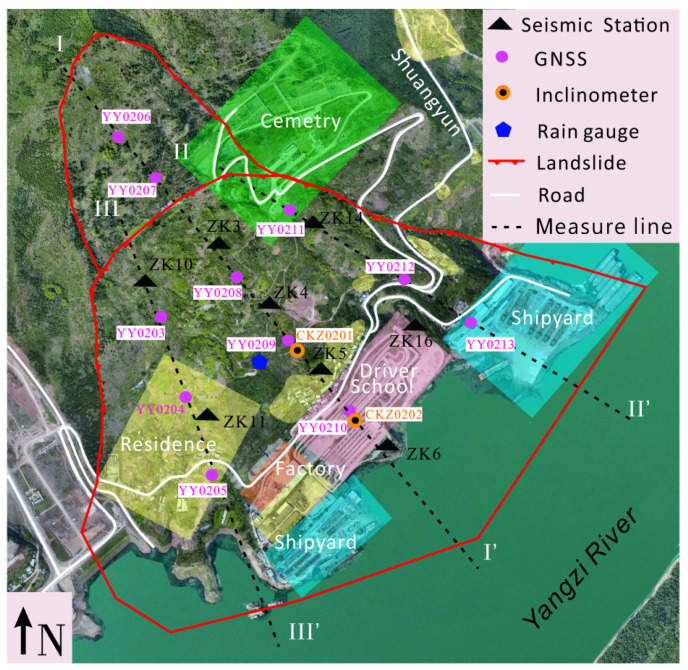
Layoutof base stations for monitoring landslides at Jiuxianping. The monitoring network of Jiuxianping landslide comprises 11 GNSS displacement sensors (YY0206, YY0207, YY0208, YY0209, YY0210 in suvery line I–I’; YY0203, YY0204, YY0205 in line II–II’; YY0211, YY0212, YY0213 in line III–III’), two inclinometers (CKZ0201, CKZ0202), eight microseismic monitoring stations (ZK3, ZK4, ZK5, ZK6, ZK10, ZK11, ZK14 and ZK16), several rain gauges, a soil stress sensor, fiber optic displacement sensors and several piezometers.

The dataset used in this experiment is derived from the Jiuxianping deep-seated landslide in Chongqing, China. The landslide is an ancient landslide, located in Yunyang County, on the left bank of the Yangtze River. The landslide plane is a “golden bell” shape, the longitudinal length is about 1200 m, and the average width is about 1200 m. The total plane area is about 1.44 km^2^, the average thickness is about 40 m, and the volume is about 5,700,104 m^3^ (see Figure 1).

We deployed a network of eight microseismic monitoring stations across the landslide area, each equipped with three channels to capture comprehensive seismic data. Utilizing these channels, we recorded the east–west, north–south, and vertical components of the microseismic signals, enabling thorough monitoring of the seismic activity within the landslide zone. Subsequently, we extracted the characteristic parameters from these microseismic signals, which were then employed as inputs for the machine learning models tasked with the classification tasks. Furthermore, we conducted statistical analyses on the microseismic events classified within the same category, thereby facilitating inference regarding the underlying nature of these events.

The dataset employed for evaluating each model encompassed a cumulative count of 4131 microseismic events spanning from January 2021 to April 2022. The STA/LTA method was used for event detection. In this paper, we set the short-time window (stw) to 0.4 s; the long-time window (ltw) to 14 s, taking into account the minimum error; Threshold 1 for event detection was set to 4, which was used to identify the start time of an event; and Threshold 2 for checking the start time of an event was set to 2, which was a lower threshold that helps to select the start time of a time accurately. The Minimum event duration (MINevent) was 0.4 s, which is the same as the length of the stw. The Minimum Interval (MINinterval) was 14 s, which was the same as the length of the ltw. The parameter MINinterval was used to separate consecutive events.

### 2.1. Features

Effective feature extraction methods can highlight the differences between different types of microseismic signals. To obtain good-quality seismic event classification, the choice of seismic features is critical. Energy will be released in the type of microseismic waves by cracks in rocks. The source parameters of microseismic events will be different depending on the fracture mode of the rocks. Based on the above characteristics, the characteristic parameters of the received microseismic events can be used as a criterion to distinguish signals [19]. During the time it was installed, the microseismic monitoring station continuously monitored the surface rock rupture and surrounding signals of the landslide in Yunyang County, Chongqing. The signals can be quantitatively represented by the feature. The expression ability of features is related to the efficiency of the accuracy rate of microseismic event recognition.

Many of the features have already worked very well in this field such as features based on signal waveform attributes and characteristic parameters based on spectrum attributes and polarity, etc., which can reflect the characteristics of the signal from a certain perspective, so that the classifier can obtain a better classification effect.

In this paper, 60 features are calculated to describe the signal in the different dimensions of the time domain, frequency domain, time and frequency domain, and multi-station network. These features were recommended by the referenced literature: Hibert et al. [20], Provost et al. [15], and Wenner et al. [21]. After a comprehensive analysis of the actual signal conditions and the specific characteristics of the research site, these features were selected. All the features are automatically calculated based on the original signal, without more artificial intervention, and only the frequency band in the Kurtosis attribute for filtering needs to be set (based on the actual situation of the signals studied in this paper, the frequency bands of 5–10 Hz, 10–50 Hz, 5–70 Hz, 50–100 Hz, and 5–100 Hz were used).

### 2.2. Manual Classification Standard

The manual classification dataset was meticulously curated to serve as a foundational resource for both the algorithmic model training and subsequent accuracy validation. Drawing inspiration from the classification criteria proposed by Langet et al. [16] in their work on automatic seismic signal classification recorded on the Åknes rock slope in Western Norway, our dataset delineates distinct seismic event classes.

**Figure 2:**
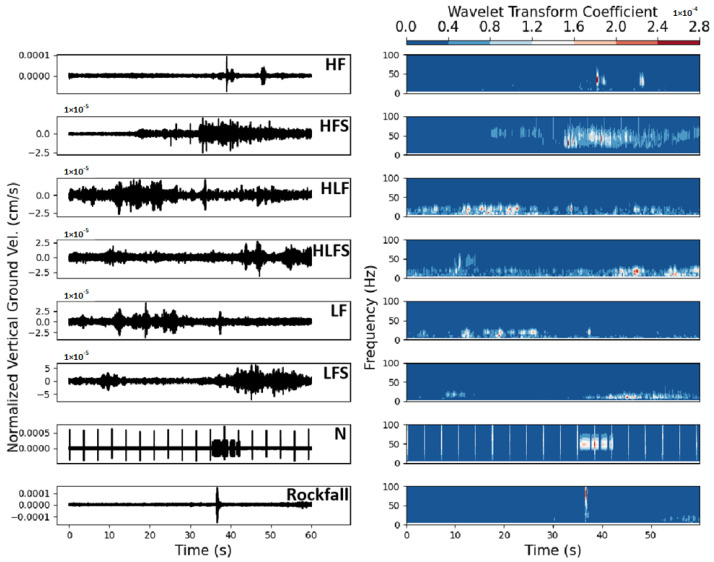
Seismic signal examples for different types of events.

The primary class of seismic events, termed slopequakes, encompasses phenomena associated with slope fracturing or sliding. This class is further stratified based on criteria such as the frequency range, duration, and peak intensity into subcategories, including high-frequency slopequakes (HF), low-frequency slopequakes (LF), the succession of high-frequency events (HFS), the succession of low-frequency events (LFS), and two specialized types denoted as dual-frequency center events (HLF and HLFS). Examples of seismic signals for different types of events are shown in Figure 2. The HLF and HLFS were found during the manual classification process. As can be seen in Figure 2, a microseismic event appears as a double frequency center, between 10–20 Hz and 20–40 Hz, respectively. The maximum frequency is generally greater than 30 Hz. They are distinguished based on their duration. Events with a duration of less than 5 s are labeled as High-Low Frequency (HLF), while events lasting more than 5 s are labeled as HLFS. Additionally, the dataset captures surface processes triggered by slope steepness and instability, such as rockfalls. Moreover, it includes a category for environmental noise (N), encompassing various sources of ambient noise. Notably, seismic events attributed to earthquakes were absent during the temporal scope of the dataset, thus warranting their exclusion from consideration. The manual classification standard can be found in Appendix Table A2.

We analyzed the waveforms and spectrograms corresponding to the microseismic events and classified them according to our manual classification criteria. The microseismic event dataset includes a total of 4131 microseismic events from January 2021 to April 2022. To uphold the dataset’s randomness and representativeness amidst seasonal and weather fluctuations, the dates and time intervals were randomly sampled.

**Figure 3:**
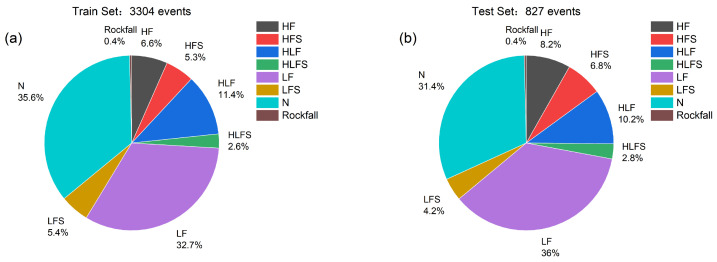
Pie diagrams showing the distribution of events within the different classes (**a**) in the training set and (**b**) in the test set, both expressed in terms of the number of events and percentages.

The dataset encompasses various categories, including N, LF, HLF, HF, HFS, LFS, HLFS, and rockfall, among others. The distribution of samples within each category is outlined in Table 1 and Figure 3, alongside the corresponding count of microseismic events per category. Due to the varying probabilities of occurrence of different microseismic events, the number of events obtained through manual processing varies. Consequently, the number of events for each type in the dataset differs. For example, RF events have a lower probability of occurrence in real situations and are therefore rarer, resulting in less data for this event type and a smaller proportion of the dataset.

## 3. Methods

Typically, human scrutiny of event data is indispensable for acquiring microseismic event classification outcomes. The training dataset, grounded in manual classification, furnishes details regarding each event alongside corresponding feature parameters. Leveraging machine learning techniques, patterns embedded within numerous parameters can be discerned, thereby facilitating the automated derivation of microseismic event classifications while curtailing human involvement. In this study, a spectrum of classification methods was employed, encompassing conventional machine learning algorithms as well as those integrating ensemble learning principles. Subsequently, we elucidate the operational mechanisms of select algorithms.

### 3.1. Non-Ensemble Learning Algorithms

#### 3.1.1. Naive Bayes

NB is grounded in Bayes’ Rule, wherein the training phase involves the estimation of prior and conditional probabilities while the testing phase computes the posterior probability for each potential category. Subsequently, the category with the highest a posteriori probability is designated as the final classification outcome, which is the prior probability of the category and is the conditional probability of observing the feature under the category. The Naive Bayes algorithm is adept at handling high-dimensional data and exhibits superior classification performance. However, it simplifies probability calculations by assuming feature independence within each category [22,23].

(1) $$P\left(C_{k}|x\right)=\frac{P\left(C_{k}\right)\cdot P\left(x|C_{k}\right)}{P(x)}$$

#### 3.1.2. Logistic Regression

LR is based on the logistic function, which estimates the probability of an event occurring by applying a linear combination of input features and model parameters to the logistic function [24]. This logistic function transforms continuous values into a range between 0 and 1, signifying the likelihood of event occurrence. Logistic regression algorithms address multi-category problems through a one-to-many strategy, designating one category as the “positive category” and the remaining -categories as “negative categories”. Each binary classifier is trained using a logistic regression model to distinguish between the positive and negative categories. During prediction, all K binary classifiers are employed to predict a new event, each returning a probability score indicating the likelihood of the data point belonging to the positive category. Subsequently, the category with the highest probability score is selected as the final classification outcome.

#### 3.1.3. Support Vector Machine

The primary objective of the SVM is to determine the optimal classification hyperplane for K feature classes, forming a K-dimensional space, which maximizes the classification margin while ensuring classification accuracy. This entails inputting feature data into the K-dimensional space to achieve category classification [25,26,27]. The classification margin represents the distance between the closest samples to the classification hyperplane and the hyperplane itself. Therefore, the problem of constructing the optimal hyperplane is translated into an optimization problem to identify the optimal solution and select the most suitable classification hyperplane. The algorithm seeks the extreme value solution, ensuring that it is a global optimal solution rather than a local minimum. This characteristic enhances the SVM algorithm’s generalization ability to unknown samples [24].

#### 3.1.4. Linear Discriminant Analysis

Linear Discriminant Analysis (LDA) is frequently employed for dimensionality reduction and feature extraction to address classification problems, effectively projecting a high-dimensional space consisting of high-dimensional data onto a lower-dimensional space through linear projection [28,29]. LDA mandates that the projected sample points achieve reduced distances between sample points belonging to the same category while ensuring that sample points from distinct categories are positioned farther apart in the projected space. This optimization is achieved by quantitatively selecting the ratio of maximizing inter-category variance to minimizing intra-category variance.

#### 3.1.5. Perceptron

Perceptron (PCT) is similar in principle to the Support Vector Machine. The aim is to find a hyperplane that separates data points of different categories. It is focused only on whether the classification is correct or not and does not consider the size of the interval. Originally designed for binary classification, Perceptron uses a one-to-many strategy to extend it to multi-category classification. The important limitation is that it only works with linearly separable data, i.e., there exists a hyperplane that perfectly separates two categories.

#### 3.1.6. Decision Tree

DT operates on a tree structure to classify microseismic events into distinct categories [30,31]. The DT model comprises nodes and edges, with each internal node denoting a feature or attribute and each leaf node representing a category or value. Through a process of recursive splitting, decision tree partitions the values of each feature into smaller subsets based on the characteristics of the training set, subsequently constructing the tree to accurately classify all the events into specific categories.

### 3.2. Ensemble Learning Algorithms

#### 3.2.1. Random Forest

RF, an ensemble learning method built upon decision trees, aggregates multiple weak decision trees through a majority vote mechanism to determine classifications. Renowned for its scalability and user-friendliness, the RF algorithm not only simplifies the classification process but also ranks the importance of features in contributing to accurate classification. Moreover, RF exhibits promising results in microseismic signal classification. Wenner et al. [21] applied the RF algorithm to detect and distinguish between slope instability, noise, and seismic signals, demonstrating its robust recognition capabilities even with limited training data. Similarly, Provost et al. [15] leveraged the RF algorithm on clay slopes, achieving a remarkable 93% improvement in recognition accuracy compared to manually classified datasets, thus highlighting RF’s efficacy in microseismic event classification.

#### 3.2.2. Gradient Boosting Trees

Similar to RF, GB also relies on decision trees, facilitating regression or classification tasks by constructing a sequence of decision tree models. Each tree is built based on the residuals of the preceding tree. GB incrementally enhances the model by iteratively adding trees, with each new tree optimized on top of all the preceding trees, thereby progressively enhancing the model accuracy [32]. The key advantage of GB lies in its robust predictive performance and ability to handle large-scale datasets and effectively model complex nonlinear relationships. Consequently, it finds extensive utility across diverse domains.

#### 3.2.3. Extreme Gradient Boosting

Extreme Gradient Boosting (XGBoost) is an adaptable and portable boosting decision tree algorithm pioneered by Chen [33]. The parallel tree boosting methodology offered by XGBoost facilitates the rapid and precise resolution of various data science challenges. XGBoost represents an enhancement over GB, boasting high flexibility and scalability to accommodate diverse data types. Moreover, it supports customized loss functions and evaluation metrics, offering abundant parameter configurations for extensive model customization.

Enabled by optimized algorithms and parallel computing, XGBoost enables swift and efficient training and prediction processes. It excels in managing large-scale datasets and high-dimensional feature spaces, demonstrating outstanding performance in numerous machine learning competitions and real-world applications.

#### 3.2.4. Light Gradient Boosting Machine

Light Gradient Boosting Machine (LGBM) is an efficient and rapid machine learning framework founded on Gradient Boosting Trees, renowned for its outstanding performance in numerous machine learning competitions.

Utilizing histogram-based techniques, LGBM effectively diminishes memory consumption and computational overhead, consequently enhancing the training speed. LGBM provides support for both column-wise and row-wise storage formats, allowing users to select the most appropriate storage scheme based on the number of features and samples, thereby further reducing memory usage.

## 4. Classification Performance

Based on the datasets constructed in this paper, microseismic events are classified using several algorithms commonly used in machine learning for multi-classification problems. The implementation of these models is realized through programming methodologies leveraging the scikit-learn library within Python, encompassing ten distinct machine learning algorithms. In our pursuit to compare the performance of varied algorithmic models, uniform parameter configurations were employed to train each model on the identical dataset.

### 4.1. Evaluation Methodology

In this investigation, model performance was comprehensively assessed using several metrics, including the accuracy, precision, recall, and F1 score. The accuracy rate denotes the ratio of correctly classified samples by the model to the total sample count. The precision rate signifies the proportion of samples predicted as positive categories that are indeed positive, serving as an indicator of the model’s true positive predictions. Recall denotes the proportion of true examples correctly identified as positive by the classifier. The F1 score amalgamates the precision and recall, representing the harmonic mean of these metrics [24].

(2) $$\begin{array}{cc} Accuracy & =\frac{TP+TN}{TP+FP+TN+FN} \end{array}$$

(3) $$\begin{array}{cc} Precision & =\frac{TP}{TP+FN} \end{array}$$

(4) $$\begin{array}{cc} Recall & =\frac{TP}{TP+FP} \end{array}$$

(5) $$\begin{array}{cc} F1-Score & =\frac{2\cdot Precision\cdot Recall}{Precision+Recall} \end{array}$$

### 4.2. Split Ratio of the Dataset

**Figure 4:**
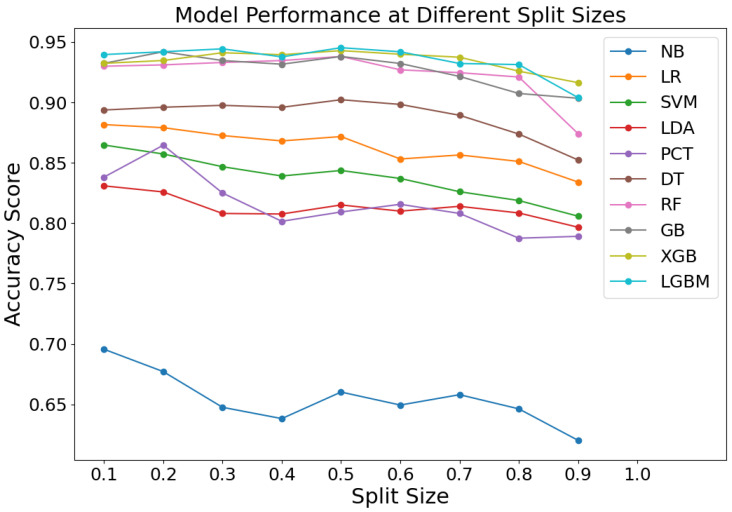
Classification accuracy with different sample sizes.

To investigate the influence of the training set size on the efficacy of machine learning algorithms, the train_test_split function from the scikit-learn package in Python was employed to partition the original dataset into training and test subsets proportionally. Considering the impact of varying training set sizes on model accuracy, increments of 10% from 10% to 90% of the total dataset were allocated to the test set for model training. The accuracy rate was selected as the evaluation metric to discern the advantages and drawbacks of different training set configurations. Figure 4 shows the performance of various machine learning models at different split sizes, with the accuracy scores plotted on the *y*-axis and the split sizes on the *x*-axis. The findings, depicted in Figure 4, reveal that the classification accuracy of the NB models is notably sensitive to changes in the training set size. LR, SVM, LDA, PCT, DT, RF, GB, XGB, and LGBM maintain stable accuracy (0.85–0.95) with minimal fluctuation. RF, GB, XGB, and LGBM consistently perform best, achieving accuracies near or above 0.9. The model is mostly able to fetch better accuracy when the partition ratio is at 0.2, 0.3, and 0.5. In order to enable more data to be involved in the training, in conjunction with the actual situation, a training set–test set ratio of 0.2 was selected. It means that the training set constitutes 80% of the total dataset with the remaining 20% for testing.

### 4.3. Validation Accuracies

**Figure 5:**
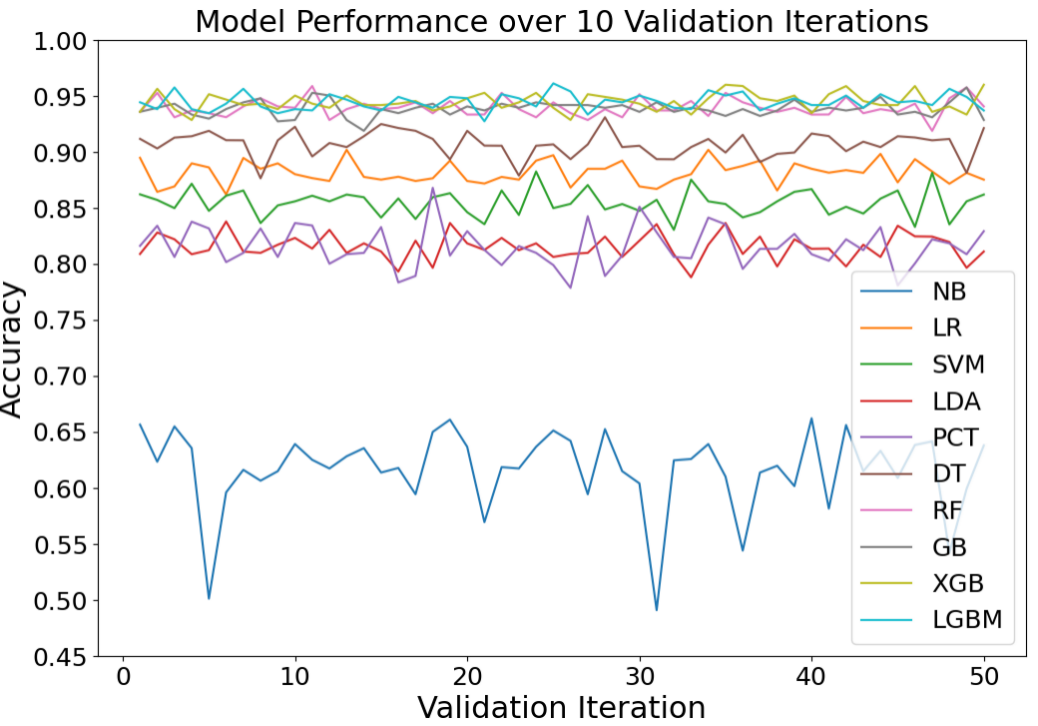
The accuracies obtained on 50 validations by different machine learning methods.

Following the determination of the training set proportions, we conducted fivefold cross-validation [34] to randomly partition the training dataset into five subsamples. This entailed training the model on four subsets while validating on the remaining subset. This process was iterated five times, with different subsets designated as the validation sets in each iteration. To assess algorithm stability, we performed 10 repetitions of fivefold cross-validation for each algorithmic model. Each run entailed the random resampling of the training data, resulting in a total of 50 validation outcomes (see Figure 5).

The algorithm stability was evaluated by computing the mean and variance across the 50 validation results (refer to Table 2). By comparing the mean accuracy and variance of the different algorithms, it is evident that GB and LGBM perform the best on this dataset, with the highest mean accuracy (94.20%) and the lowest variance (0.49 and 0.52, respectively). RF and XGB follow closely, also showing high accuracy and low variance. In contrast, NB performs the worst, with the lowest accuracy and highest variance. Overall, the ensemble learning algorithms (such as GB, LGBM, RF, and XGB) outperform the traditional machine learning algorithms on this dataset.

**Figure 6:**
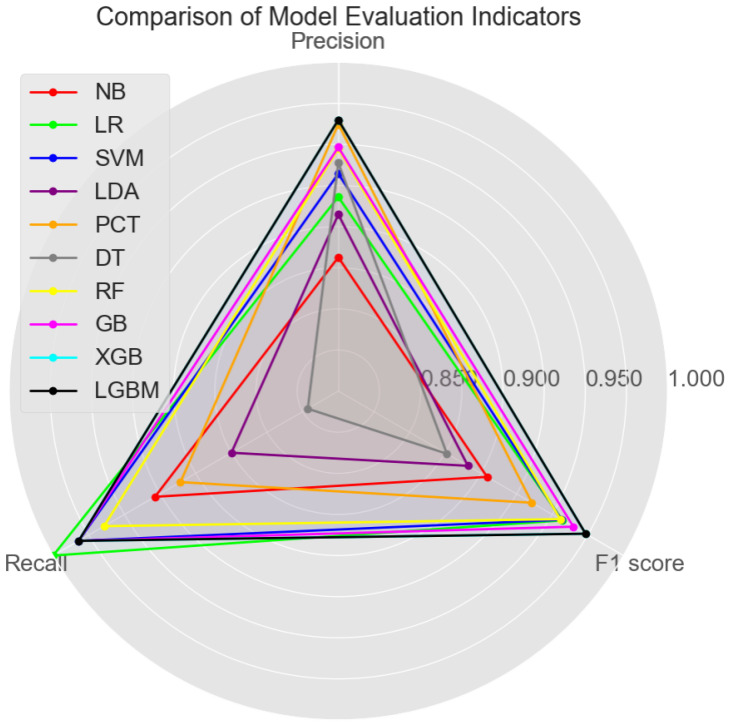
Comparison of different classifiers on each evaluation indicator.

In the evaluation of machine learning models, multiple metrics are commonly used to comprehensively measure the performance of the model. Common evaluation metrics for classification models include the accuracy, precision, and recall. To visualize the combined performance of these metrics, a radar chart can be used. The average values of the precision, recall, and F1 score for the model over 50 cross-validations are shown in Figure 6. Each evaluation metric is represented as a dimension, forming a polygon. The area of the polygon intuitively reflects the model’s overall performance across all metrics: the larger the area, the better the model performs on the evaluation metrics.

In terms of comprehensive performance, XGB and LGBM achieve the highest F1 score of 0.973, as well as the highest precision (0.965) and recall (0.982). RF and GB follow closely, with F1 scores of 0.956 and 0.965, respectively. Although slightly behind XGB and LGBM, both models exhibit relatively high precision and recall. Among the other models, SVM and LR also show strong performance, particularly in accuracy and F1 scores. In contrast, NB and LDA demonstrate relatively lower performance. While they excel in recall, their combined performance in terms of the precision and F1 score does not match the higher-performing models, especially concerning the F1 score. These results underscore the superior performance metrics of ensemble learning models compared to non-ensemble models. The polygonal representation in Figure 6 provides a clear visual comparison, further highlighting the overall effectiveness of ensemble methods in classification tasks.

### 4.4. Category Performance

**Figure 7:**
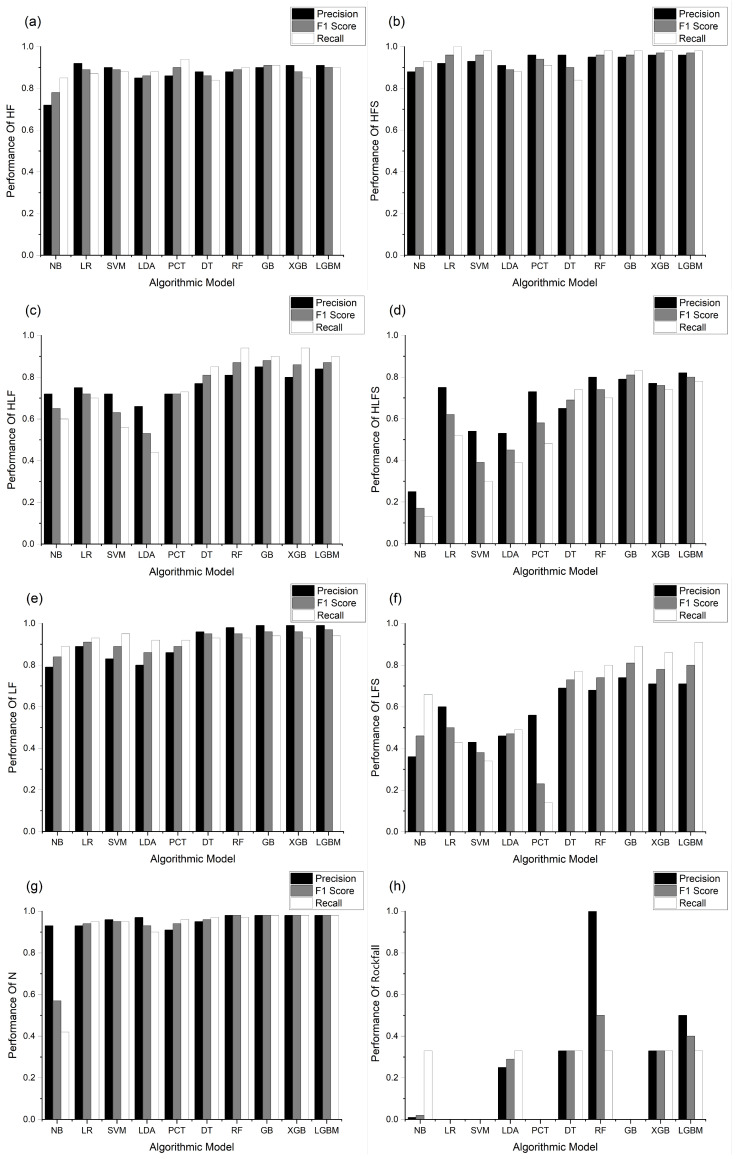
Performance of classification models implemented by different AI algorithms on different types of datasets. Black, gray, and white represent accuracy, F1 score, and recall evaluation metrics, respectively. (**a**–**h**) represent the classification results of each model on categories HF, HFS, HLF, HLFS, LF, LFS, N, and Rockfall, respectively.

The performance of models varies across different event types. Specifically, in the evaluation of the precision, the microseismic events were categorized into eight types: HF, HFS, HLF, HLFS, LF, LFS, N, and rockfall. For each event type, assessments were conducted using three evaluation metrics: precision, F1 score, and recall (refer to Figure 7).

Across the spectrum of the ensemble learning algorithms, superior performance is observed for each event type. As can be seen from the figure, out of the given eight types, the four types HF, HFS, LF and N get more similar results in various algorithms and are able to achieve better classification results. Conversely, the event types HLF, HLFS, and LFS demonstrate an enhanced precision, F1 score, and recall compared to the other algorithms, showcasing the effectiveness of the ensemble learning algorithms in their classification. Notably, the rockfall event type may not fully reflect the classification effectiveness during testing due to its limited event count.

**Figure 8:**
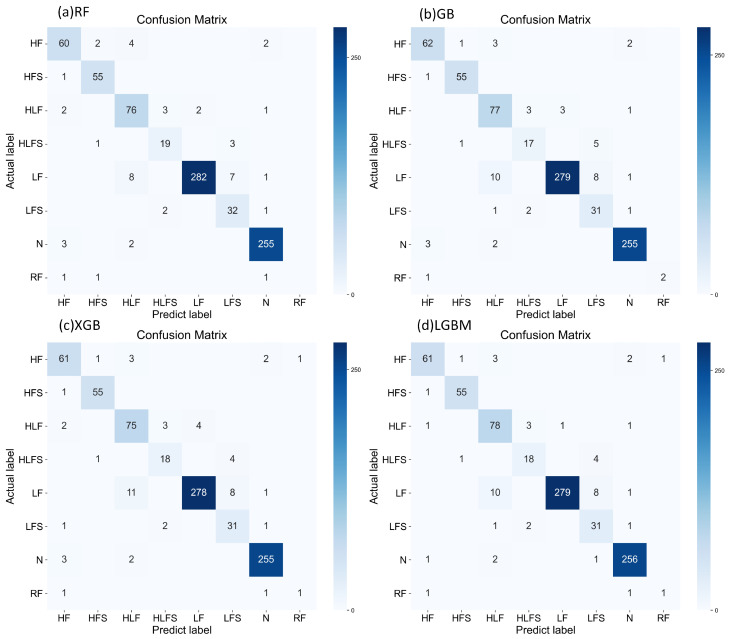
Confusion matrices for the four models of ensemble learning.

Meanwhile, Figure 8 is shown, which contains the confusion matrices for the four models of ensemble learning: (a) RF, (b) GB, (c) XGB, and (d) LGBM. These confusion matrices demonstrate the specific performance of each model in the classification task, including the number of correct and incorrect classifications. It further demonstrates their effectiveness in real-world applications.

### 4.5. Model Optimization

Grid search and cross-validation were used to optimize the parameters of the model [35]. Grid search systematically explores all possible combinations of parameters by evaluating each combination through cross-validation to select the best parameters. Firstly, we listed the hyperparameters for each model to adjust their possible ranges. Subsequently, we generated all possible parameter combinations. For each combination, we evaluated the model using cross-validation, splitting the dataset into multiple subsets and training and validating the model multiple times to assess the stability and performance of each parameter combination. Finally, based on the cross-validation results, we selected the parameter combination that provided the best performance. Selecting the optimal parameter values for the random forest algorithm resulted in a 0.73% improvement in model accuracy. In XGB, the algorithmic accuracy witnessed a enhancement of 0.12% following the parameter adjustments. Similarly, within LGBM, the optimization efforts involved modifying model parameters such as n_estimators, learning_rate, max_depth, num_leaves, and min_child_samples, alongside the inclusion of regularization parameters, namely, class_weight, reg_alpha, and reg_lambda, aimed at mitigating overfitting. Regularization parameters help us control the complexity of the model. They achieve this by enhancing the influence of key features while effectively reducing the weights of less relevant features in the model. As a result, the model’s accuracy increased from 93.95% to 94.70%. The specific parameter values and tuning ranges are detailed in Table 3.

### 4.6. Application

The monitoring station continuously observed the landslide site from 1 May 2021 to 30 June 2022. The data collected during this interval underwent predictions utilizing the previously established classification model. The relative fluctuations in the event counts are depicted in Figure 8.

**Figure 9:**
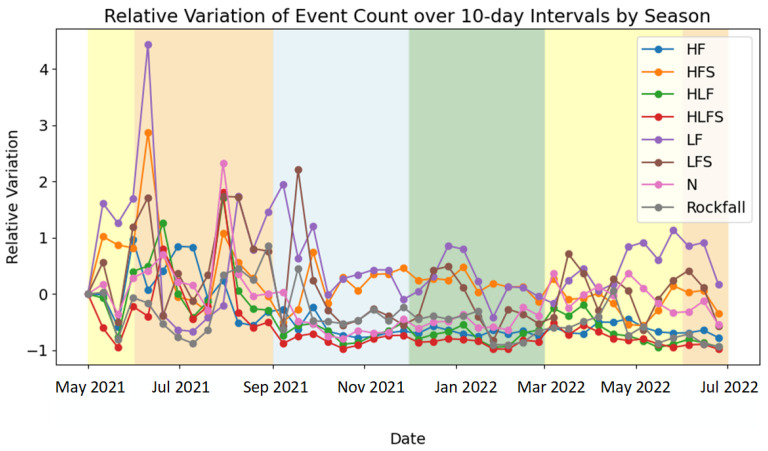
Relative Variation in the number of events N per class over chunks of 10 days. The reference value is taken from the first chunk.

The relative change R in the number of events N in each category over a 10-day time period is computed in Figure 9.

(6) $$R=\frac{N-N(1)}{N(1)}$$

As depicted in the figure, the HF events exhibit greater variability during the initial placement of the instruments in the summer months, followed by a decrease in occurrences in the subsequent year. The HFS events demonstrate consistent characteristics, with a notable surge in the event count during the initial ten days of June, followed by minimal variation throughout the year, maintaining levels similar to those observed during the initial period. Both the HLF and HLFS events exhibit elevated event counts during June and August, followed by a decrease in July and the subsequent autumn, winter, and spring seasons. The LF events peak in mid-June with a subsequent sharp decline over the following month, maintaining relatively consistent levels throughout the year. Similarly, the LFS events peak in mid-June and remain stable thereafter. The occurrence of N events is comparatively low, with consistent characteristics throughout the year. The LF events consistently peak in mid-June, experiencing a sudden decline in the subsequent month, after which they stabilize. The LFS events demonstrate higher event counts during the summer months. The rockfall events display heightened occurrences in the late summer and early autumn months. Notably, the LF, HFS, and LFS event types all exhibit a sudden surge during the mid-June period, potentially influenced by the prevailing weather conditions at that time.

## 5. Conclusions

This paper employs ten machine learning methodologies to classify microseismic events through the construction of a microseismic event dataset. When employing default parameters, DT exhibits the highest performance among the non-integrated learning-based algorithms, achieving an accuracy of 88.75%. The models based on ensemble learning—RF, GB, XGB, and LGBM—demonstrate superior performance, ranging between 93.5% and 94.2% accuracy. Taking into account the precision, recall, and F1 score, XGB and LGBM are the better choices. It is suitable for tasks that require a balance between precision and recall. GB and RF also perform well but slightly less well than XGB and LGBM. These experimental outcomes underscore the efficacy of ensemble learning in enhancing model robustness by amalgamating predictions from multiple base models, thereby mitigating over-reliance on individual models and diminishing the risk of overfitting.

Furthermore, specific classification experiments were conducted for each microseismic event category. The results reveal a comparable performance between non-ensemble and ensemble learning models across categories, such as HF, HFS, HLF, LF, and N. However, a more pronounced disparity between the two models is observed in the classification of the microseismic categories HLFS and LFS, suggesting inherent difficulties in distinguishing these categories from each other.

The ratio of the training set to the test set impacts model performance, with optimal results achieved at an 8:2 ratio. Additionally, parameter tuning substantially influences model training outcomes. Employing a grid search methodology facilitates the identification of optimal model parameters compared to empirical settings. In light of the rapid advancements in artificial intelligence, our future endeavors will focus on exploring more efficient classification models to continually enhance the accuracy of microseismic event classification.

## Figures and Tables

**Table 1:** The number of events in the train and test sets.

Type of Events	Train Set	Test Set
HF	219	68
HFS	176	56
HLF	376	84
HLFS	86	23
LF	1082	298
LFS	177	35
N	1175	260
Rockfall	13	3

**Table 2:** Mean of accuracy and variance of different machine learning algorithm.

Machine Learning Algorithm	Mean of Accuracy (%)	Variance
NB	67.71	12.9
LR	87.91	1.01
SVM	85.73	1.39
LDA	82.59	1.31
PCT	86.46	3.43
DT	88.75	1.29
RF	93.59	0.63
GB	94.20	0.49
XGB	93.47	0.62
LGBM	94.20	0.52

**Table 3:** Tabel of model parameter tuning.

Model	Parameter	Range	Optimal Value
RF	n_estimators	(1, 1000)	122
	max_depth	(1, 40)	16
	min_samples_leaf	(1, 40)	1
	min_samples_split	(2, 40)	19
	max_features	(1, 40)	33
	criterion	[“gini”, “entropy”]	“gini”
	class_weight	[None, “balanced”]	“balanced”
GB	n_estimators	(1, 1000)	263
	learning_rate	[0.1, 0.5, 0.01]	0.1
	max_depth	(1, 40)	9
	min_samples_split	(2, 40)	12
	min_samples_leaf	(1, 40)	17
XGB	n_estimators	(1, 1000)	61
	learning_rate	[0.1, 0.5, 0.01]	0.15
	max_depth	(1, 40)	6
	min_child_weight	(1, 10)	5
	gamma	[0.1, 0.2, 0.3, 0.4, 0.5, 0.6, 0.7, 0.8, 0.9, 1.0]	0.2
	subsample	[0.1, 0.2, 0.3, 0.4, 0.5, 0.6, 0.7, 0.8, 0.9, 1.0]	1.0
	colsample_bytree	[0.1, 0.2, 0.3, 0.4, 0.5, 0.6, 0.7, 0.8, 0.9, 1.0]	1.0
	reg_alpha	[0, 0.1, 0.3, 0.5, 0.7, 0.9, 1.0, 1 × 10^−2^, 1 × 10^−3^, 1 × 10^−4^, 1 × 10^−5^]	1 × 10^−4^
LGBM	n_estimators	(1, 1000)	131
	learning_rate	[0.1, 0.5, 0.01]	0.1
	max_depth	(1, 40)	18
	num_leaves	(1, 40)	31
	min_child_samples	(1,40)	20
	reg_alpha	[0, 0.1, 0.3, 0.5, 0.7, 0.9, 1.0, 1 × 10^−2^, 1 × 10^−3^, 1 × 10^−4^, 1 × 10^−5^]	1 × 10^−3^
	reg_lambda	[0, 0.1, 0.3, 0.5, 0.7, 0.9, 1.0, 1 × 10^−2^, 1 × 10^−3^, 1 × 10^−4^, 1 × 10^−5^]	1 × 10^−5^

## Data Availability

The data presented in this study are available on request from the corresponding author due to research as part of the Natural Science Foundation of Jiangxi Province.

## Appendix A

In Table A1 below, the features used to train the classification model are presented. Subsequently, the manual classification standards are shown in Table A2.

**Table A1:** The features able to represent the signals.

	Description	Formula	Sources
1	Maximum amplitude of the signal	$A_{m}=\max\left(x_{i}\right)$	Feng et al. [1]
2	Maximum frequency of the signal	$F_{m}=\max\left(FFTA_{i}\right)$	Feng et al. [1]
3	Energy distribution between the high-frequency bands [20–100 Hz] and low-frequency bands [3–16 Hz]	$\frac{\sum_{i=20}^{100}FFTA_{i}^{2}}{\sum_{i=3}^{16}FFTA_{i}^{2}}$	Feng et al. [1]
4	Centroid frequency	$\gamma =\frac{m_{2}}{m_{1}}$, with $m_{1}$ and $m_{2}$: first and second moment	Provost et al. [15]
5	Duration	$D=t_{f}-t_{i}$, with $t_{i}$ and $t_{f}$: beginning and end of the signal	Provost et al. [15]
6	Energy	$E=\sum_{i=1}^{N}x_{i}^{2}$	Feng et al. [1]
7	Energy density	$E_{a}=\frac{\sum_{i=1}^{N}x_{i}^{2}}{t_{f}-t_{i}}$	Feng et al. [1]
8	Energy variation	$\frac{E_{a}}{A_{m}^{2}}$	Feng et al. [1]
9	Number of peaks	-	Feng et al. [1]
10	Ratio of the mean over the maximum of the envelope signal	-	Provost et al. [15]
11	Ratio of the median over the maximum of the envelope signal	-	Provost et al. [15]
12	Ratio between the ascending and descending time	$\frac{t_{max}-t_{i}}{t_{f}-t_{max}}$, with $t_{max}$: time of the largest amplitude	Provost et al. [15]
13	Kurtosis of the raw signal (peakness of the signal)	$\frac{m_{4}}{\sigma^{4}}$, with $m_{4}$: fourth moment, σ: standard deviation	Provost et al. [15]
14	Skewness of the raw signal	$\frac{m_{3}}{\sigma^{3}}$, with $m_{3}$: third moment	Provost et al. [15]
15	Kurtosis of the envelope	see 13	Provost et al. [15]
16	Skewness of the envelope	see 14	Provost et al. [15]
17	Energy in the first third part of the autocorrelation function	$\int_{0}^{\frac{T}{3}}C\left(\tau \right)d\tau$, with T: signal duration, *C*: autocorrelation function	Provost et al. [15]
18	Energy in the remaining part of the autocorrelation function	see 17	Provost et al. [15]
19	Ratio of 17 and 18	-	Provost et al. [15]
20	Number of peaks in the autocorrelation function	-	Provost et al. [15]
21–25	Energy of the signal filtered in the 5–10 Hz, 10–50 Hz, 5–70 Hz, 50–100 Hz, and 5–100 Hz	$\int_{0}^{T}y_{f}\left(t\right)dt$, with $y_{f}$: filtered signal in the frequency range [$f_{1}-f_{2}$]	Provost et al. [15]
26–30	Kurtosis of the signal in the 5–10 Hz, 10–50 Hz, 5–70 Hz, 50–100 Hz, and 5–100 Hz frequency range	see 21–25	Provost et al. [15]
31	Decreasing part of the signal and $I\left(t\right)=Y_{max}-\frac{Y_{max}}{t_{f}-t_{max}}t$	$\sqrt{\bar{Y\left(t\right)-I{\left(t\right)}^{2}}}$,with Y: envelope of the signal	Provost et al. [15]
32	Mean of the DFT	DFT: discrete Fourier transform mean(abs(fft(Zdata)))	Hibert et al. [20]
33	Central frequency of the 1st quartile	-	Wenner et al. [21]
34	Central frequency of the 2nd quartile	-	Wenner et al. [21]
35	Median of the normalized DFT	-	Hibert et al. [20]
36	Variance of the normalized DFT	-	Hibert et al. [20]
37	Energy in [0, $\frac{1}{4}$]N $y_{f}$	$\int_{f_{1}}^{f_{2}}DFT\left(f\right)df$, with $f_{1},f_{2}$: considered frequency range	Hibert et al. [20]
38	Energy in [$\frac{1}{4}$, $\frac{1}{2}$]N $y_{f}$	-	Hibert et al. [20]
39	Energy in [$\frac{1}{2}$, $\frac{3}{4}$]N $y_{f}$	-	Hibert et al. [20]
40	Energy in [$\frac{3}{4}$, 1]N $y_{f}$	-	Hibert et al. [20]
41	Number of peaks	-	Feng et al. [36]
42	Gyration radius	$\gamma_{2}=\sqrt{\frac{m_{3}}{m_{2}}}$, with $m_{3}$: third moment	Provost et al. [15]
43	Kurtosis of the maximum of all discrete Fourier transforms (DFTs) as a function of time *t*	$Kurtosis[\max_{t=0,\ldots ,T}\left(SPEC\left(t,f\right)\right)]$, with SPEC(t,f): spectrogram	Provost et al. [15]
44	Mean ratio between the maximum and the mean of all DFTs	$Mean(\frac{\max(SPEC)}{mean(SPEC)})$	Provost et al. [15]
45	Number of peaks in the curve showing the temporal evolution of the DFTs maximum	-	Provost et al. [15]
46	Number of peaks in the curve showing the temporal evolution of the DFT mean	-	Provost et al. [15]
47	Number of peaks in the curve showing the temporal evolution of the DFT median	-	Provost et al. [15]
48	Number of peaks in the curve of the temporal evolution of the DFT central frequency	-	Provost et al. [15]
49	Number of peaks in the curve of the temporal evolution of the DFT maximum frequency	-	Provost et al. [15]
50	Ratio between 45 and 46	-	Provost et al. [15]
51	Ratio between 45 and 47	-	Provost et al. [15]
52	Mean ratio between the maximum and the median of all DFTs	$Mean(\frac{\max(SPEC)}{Median(SPEC)})$	Provost et al. [15]
53	Ratio between 48 and 49	-	Provost et al. [15]
54	Mean distance between the curves of the temporal evolution of the DFT maximum frequency and mean frequency	-	Provost et al. [15]
55	Mean distance between the curves of the temporal evolution of the DFT maximum frequency and median frequency	-	Provost et al. [15]
56	Mean distance between the 1st quartile and the median of all DFTs as a function of time	-	Provost et al. [15]
57	Mean distance between the 3rd quartile and the median of all DFTs as a function of time	-	Provost et al. [15]
58	Mean distance between the 3rd quartile and the 1st quartile of all DFTs as a function of time	-	Provost et al. [15]
59	The ratio of 2 between two different seismic stations	-	Feng et al. [1]
60	The ratio of 1 between two different seismic stations	-	Feng et al. [1]

**Table A2:** Manual classification standard.

Class	Sub-Class	Duration	Frequency	Other	Potential Origin
Slopequake (at depth)	High Frequency (HF)	<5	30–60 Hz, Max_frequency > 30 Hz	Very impulsive	Rock break, crack open
	Low Frequency (LF)	<5	4–20 Hz	-	Soil shearing on sliding plane
	High-Low Frequency (HLF)	<5	Double center: one 10–25 Hz, another 20–40 Hz, Max_frequency > 15 Hz	-	Soil-rock shear on sliding plane
	Succession of HF (HFS)	>5	30–60 Hz, Max_frequency > 30 Hz	Several bursts of energy	Soil-rock shear on sliding plane
	Succession of LF (LFS)	>5	4–20 Hz	Several bursts of energy	Soil shearing on plane
	HLFS	>5	Double center: one 10–25 Hz, another 20–40 Hz	Max_frequency > 15 Hz	-
Rockfall	-	-	Large bandwidth, up to 100 Hz	The duration depends on the trajectory	-
Earthquake	-	>10	<20 Hz	-	-
Noise	-	-	<4 Hz and large bandwidth up to 250 Hz	-	-

## References

[1] Feng L., Pazzi V., Intrieri E., Gracchi T., Gigli G. (2020). Joint detection and classification of rockfalls in a microseismic monitoring network. Geophys. J. Int..

[2] Zaruba Q., Mencl V. (2014). Landslides and Their Control.

[3] Dai F., Jiang P., Xu N.W., Zhou Z., Sha C., Guo L. (2016). Study of microseismicity and its time-frequency characteristics of abutment rock slope during impounding period. Rock Soil Mech..

[4] Hardy H.R. (2003). Acoustic Emission/Microseismic Activity: Principle.

[5] Lou M., Rial J. (1995). Application of the wavelet transform in detecting multiple events of microearthquake seismograms. Geophys. Res. Lett..

[6] Allen R.V. (1978). Automatic earthquake recognition and timing from single traces. Bull. Seismol. Soc. Am..

[7] Cieplicki R., Eisner L., Mueller M. Microseismic event detection: Comparing P-wave migration with P-and S-wave crosscorrelation. Proceedings of the SEG International Exposition and Annual Meeting.

[8] Vrieze S.I. (2012). Model selection and psychological theory: A discussion of the differences between the Akaike information criterion (AIC) and the Bayesian information criterion (BIC). Psychol. Methods.

[9] Yang H., Zhu L., Chu R. (2009). Fault-plane determination of the 18 April 2008 Mount Carmel, Illinois, earthquake by detecting and relocating aftershocks. Bull. Seismol. Soc. Am..

[10] Li W., Narvekar N., Nakshatra N., Raut N., Sirkeci B., Gao J. Seismic data classification using machine learning. Proceedings of the 2018 IEEE Fourth International Conference on Big Data Computing Service and Applications (BigDataService).

[11] Lindenbaum O., Rabin N., Bregman Y., Averbuch A. Multi-channel fusion for seismic event detection and classification. Proceedings of the 2016 IEEE International Conference on the Science of Electrical Engineering (ICSEE).

[12] Zhao G., Huang H.M., Lu X.X. Discriminating earthquakes and explosion events by seismic signals basing on BP-Adaboost classifier. Proceedings of the 2016 2nd IEEE International Conference on Computer and Communications (ICCC).

[13] Astuti W., Akmeliawati R., Sediono W., Salami M.J.E. (2014). Hybrid technique using singular value decomposition (SVD) and support vector machine (SVM) approach for earthquake prediction. IEEE J. Sel. Top. Appl. Earth Observ. Remote Sens..

[14] Long Y., Chen T.X., Xu S.D. (2022). Recognition of mining rock fracture signal based on waveform feature and decision tree classification algorithm. China Min. Mag..

[15] Provost F., Hibert C., Malet J.P. (2017). Automatic classification of endogenous landslide seismicity using the Random Forest supervised classifier. Geophys. Res. Lett..

[16] Langet N., Silverberg F.M.J. (2023). Automated classification of seismic signals recorded on the Åknes rockslope, Western Norway, using a Convolutional Neural Network. Earth Surf. Dyn..

[17] Malfante M., Dalla Mura M., Métaxian J.P., Mars J.I., Macedo O., Inza A. (2018). Machine learning for volcano-seismic signals: Challenges and perspectives. IEEE Signal Process. Mag..

[18] Maggi A., Ferrazzini V., Hibert C., Beauducel F., Boissier P., Amemoutou A. (2017). Implementation of a Multistation Approach for Automated Event Classification at Piton de la Fournaise Volcano. Seismol. Res. Lett..

[19] Peng K., Tang Z., Dong L.J., Sun D.Y. (2021). Machine learning based identification of microseismic signals using characteristic parameters. Sensors.

[20] Hibert C., Malet J.P., Bourrier F., Provost F., Berger F., Bornemann P., Tardif P., Mermin E. (2017). Single-block rockfall dynamics inferred from seismic signal analysis. Earth Surf. Dynam..

[21] Wenner M., Hibert C., Meier L., Walter F. (2020). Near real-time automated classification of seismic signals of slope failures with continuous random forests. Nat. Hazard. Earth Sys..

[22] Saritas M.M., Yasar A. (2019). Performance analysis of ANN and Naive Bayes classification algorithm for data classification. Int. J. Intell. Syst. Appl. Eng..

[23] Snoek J., Larochelle H., Adams R.P. (2012). Practical bayesian optimization of machine learning algorithms. Adv. Neural Inf. Process. Syst..

[24] Abdalzaher M.S., Moustafa S.S.R., Abd-Elnaby M., Elwekeil M. (2021). Comparative performance assessments of machine-learning methods for artificial seismic sources discrimination. IEEE Access.

[25] Osuna E., Freund R., Girosi F. (1997). An improved training algorithm for support vector machines. Neural Networks for Signal Processing VII. 1997 IEEE Signal Processing Society Workshop, Amelia Island, FL, USA, 21–23 July 1997.

[26] QI H.N. (2004). Support vector machines and application research overview. Comput. Eng..

[27] Noble W.S. (2006). What is a support vector machine?. Nat. Biotechnol..

[28] Martinez A.M., Kak A.C. (2001). Pca versus lda. IEEE Trans. Pattern Anal. Mach. Intell..

[29] Jelodar H., Wang Y.L., Yuan C., Feng X., Jiang X.H., Li Y.C., Zhao L. (2019). Latent Dirichlet allocation (LDA) and topic modeling: Models, applications, a survey. Multimed. Tools Appl..

[30] Cramer G.M., Ford R.A., Hall R.L. (1976). Estimation of toxic hazard—A decision tree approach. Food Cosmet. Toxicol..

[31] Song Y.Y., LU Y. (2015). Decision tree methods: Applications for classification and prediction. Shanghai Arch. Psychiatry.

[32] Ma X.L., Ding C., Luan S., Wang Y., Wang Y.P. (2017). Prioritizing influential factors for freeway incident clearance time prediction using the gradient boosting decision trees method. IEEE Trans. Intell. Transp. Syst..

[33] Chen T.Q., Guestrin C. Xgboost: A scalable tree boosting system. Proceedings of the 22nd ACM SIGKDD International Conference on Knowledge Discovery and Data Mining, KDD ’16: The 22nd ACM SIGKDD International Conference on Knowledge Discovery and Data Mining.

[34] Kohavi R. A study of cross-validation and bootstrap for accuracy estimation and model selection. Proceedings of the 1995 International Joint Conference on AI, Palais de Congres.

[35] Wen B., Dong W.H., Xie W.J., Jun M. (2018). Parameter optimization method for random forest based on improved grid search algorithm. Comput. Eng. Appl..

[36] Feng L., Pazzi V., Intrieri E., Gracchi T., Gigli G. (2019). Rockfall seismic features analysis based on in situ tests: Frequency, amplitude, and duration. J. Mt. Sci..

